# Severe acute respiratory infection in children in a densely populated urban slum in Kenya, 2007–2011

**DOI:** 10.1186/s12879-015-0827-x

**Published:** 2015-02-25

**Authors:** Robert F Breiman, Leonard Cosmas, M Kariuki Njenga, John Williamson, Joshua A Mott, Mark A Katz, Dean D Erdman, Eileen Schneider, M Steven Oberste, John C Neatherlin, Henry Njuguna, Daniel M Ondari, Kennedy Odero, George O Okoth, Beatrice Olack, Newton Wamola, Joel M Montgomery, Barry S Fields, Daniel R Feikin

**Affiliations:** Global Disease Detection Division and Influenza Division of the United States Centers for Disease Control and Prevention (CDC Kenya office), Nairobi, Kenya; Global Disease Detection Branch, Division of Global Health Protection, Center for Global Health, Centers for Disease Control and Prevention, Atlanta, GA USA; Division of Viral Diseases, National Center for Immunizations and Respiratory Diseases, Centers for Disease Control and Prevention, Atlanta, GA USA; The Kenya Medical Research Institute (KEMRI), Nairobi, Kenya; Current address: Emory Global Health Institute, Emory University, 1599 Clifton Road, Atlanta, GA 30322 USA

## Abstract

**Background:**

Reducing acute respiratory infection burden in children in Africa remains a major priority and challenge. We analyzed data from population-based infectious disease surveillance for severe acute respiratory illness (SARI) among children <5 years of age in Kibera, a densely populated urban slum in Nairobi, Kenya.

**Methods:**

Surveillance was conducted among a monthly mean of 5,874 (range = 5,778-6,411) children <5 years old in two contiguous villages in Kibera. Participants had free access to the study clinic and their health events and utilization were noted during biweekly home visits. Patients meeting criteria for SARI (WHO-defined severe or very severe pneumonia, or oxygen saturation <90%) from March 1, 2007-February 28, 2011 had blood cultures processed for bacteria, and naso- and oro- pharyngeal swabs collected for quantitative real-time reverse transcription polymerase chain reaction testing for influenza viruses, parainfluenza viruses (PIV), respiratory syncytial virus (RSV), adenovirus, and human metapneumovirus (hMPV). Swabs collected during January 1, 2009 – February 28, 2010 were also tested for rhinoviruses, enterovirus, parechovirus, *Mycoplasma pneumoniae*, and *Legionella* species. Swabs were collected for simultaneous testing from a selected group of control-children visiting the clinic without recent respiratory or diarrheal illnesses.

**Results:**

SARI overall incidence was 12.4 cases/100 person-years of observation (PYO) and 30.4 cases/100 PYO in infants. When comparing detection frequency in swabs from 815 SARI cases and 115 healthy controls, only RSV and influenza A virus were significantly more frequently detected in cases, although similar trends neared statistical significance for PIV, adenovirus and hMPV. The incidence for RSV was 2.8 cases/100 PYO and for influenza A was 1.0 cases/100 PYO. When considering all PIV, the rate was 1.1 case/100 PYO and the rate per 100 PYO for SARI-associated disease was 1.5 for adenovirus and 0.9 for hMPV. RSV and influenza A and B viruses were estimated to account for 16.2% and 6.7% of SARI cases, respectively; when taken together, PIV, adenovirus, and hMPV may account for >20% additional cases.

**Conclusions:**

Influenza viruses and RSV (and possibly PIV, hMPV and adenoviruses) are important pathogens to consider when developing technologies and formulating strategies to treat and prevent SARI in children.

## Background

Reducing the substantial public health burden of acute respiratory infection in children in Africa remains a major priority and an immense challenge [1,2]. Despite steady advances in characterizing principal etiologies, incidence, and factors contributing to severe respiratory infection [3,4], knowledge gaps persist [5]. Filling these gaps is critical to ensuring that limited available public health resources can be optimally targeted towards feasible, effective interventions. As has been the case for decades [6,7], pneumonia remains a major killer of children in Africa [8,9]. In 2008, it was estimated that 35 million cases of pneumonia occur per year in children <5 years old in Africa [3]; it was the cause of 18% of deaths among African children, resulting in >750,000 deaths [1].

Many of the formative studies on pneumonia etiology that provided evidence contributing to design of respiratory disease interventions, were conducted over 20 years ago [10,11]. Much has changed over the past two decades. The epidemiology of predisposing conditions for pneumonia, such as malaria, HIV and malnutrition, as well as socioeconomic status, is changing in Africa [12,13]. Recent introductions of conjugate vaccines for the two leading bacterial causes of pneumonia, *Haemophilus influenzae* type B (Hib) and *Streptococcus pneumoniae*, into the Expanded Programme on Immunization (EPI) in Africa will likely, by reducing the numbers of pneumonias that they cause, also lead to a shifted set of priority pathogens targeted for pneumonia prevention. In addition, new diagnostic technologies, including the advent of more readily available, highly sensitive molecular diagnostics, have enhanced the ability to detect respiratory pathogens [14,15]. Finally, with massive urbanization in Africa and advent of densely populated informal settlements or slums [16], different respiratory pathogen transmission patterns [17], co-morbidities, and access to health care [18,19] must be considered when comparing with sparsely populated rural areas from where most data on pneumonia epidemiology and etiology have been collected.

We recently published pneumonia burden and etiology data from population-based surveillance in rural western Kenya undertaken from 2007–2010 [20]. To broaden the knowledge-base and compare etiology and epidemiology, we analyzed data from our population-based infectious disease surveillance (PBIDS) site in Kibera, an urban slum in Nairobi. The rural and urban PBIDS operate with the same study protocol.

## Methods

### Study site

CDC and the Kenya Medical Research Institute (KEMRI) have conducted PBIDS since late 2005 in Kibera, an impoverished informal settlement in Nairobi, Kenya with population density of 77,000 people per km^2^ and suboptimal hygiene and sanitation [21,22]. All households in 2 villages, Gatwikera and Soweto West were offered enrollment. The mean surveillance population for children <5 years old was 5,874 (range = 5,778-6,411) children per month from 2007–2011. Enrollment was continuous since the project’s beginning. The under-5 mortality rate was 15.1 per 1,000 person-years of observation from 2007–2010 [23]. HIV prevalence in the surveillance area is high (14.9% in adults ≥18 years in 2008) [24]. Pneumococcal conjugate vaccine was not yet introduced during this study period, and vaccine coverage rates for diphtheria-pertussis-tetanus vaccine (as part of a pentavalent vaccine) were not available for the study area, but were felt to range between 60-80% during the study period.

### Clinic surveillance

Since March 1, 2007, all enrolled participants received free medical care for acute illnesses from KEMRI/CDC-trained clinical staff at Tabitha Clinic (owned by Carolina for Kibera) located ≤1 km from all surveillance households. Tabitha Clinic sees between 90 and 200 out-patients/day and refers all patients requiring hospitalization; it does not have in-patient facilities. Chest radiography was not available through most of the study period.

The clinic-based case definition for SARI for children <5 years old used a modification of the WHO Integrated Management of Childhood Illness algorithm for severe and very severe pneumonia, defined as a child with cough or difficulty breathing and any of the following symptoms or signs: unable to drink/breastfeed, vomits everything, convulsions, lethargic or unconscious, stridor when calm, and lower chest wall indrawing [25,26], as well as an additional criterion of oxygen saturation <90%. Blood cultures and nasopharyngeal and oropharyngeal swabs (polyester-tip) were collected [27] from SARI cases, meeting the above case definition; however, specimens were not collected from a substantial proportion of patients during periods of under-staffing, high patient volume, or if parents/caretakers did not want to have specimens obtained. Clinical differences by age group between children with SARI who were swabbed and not swabbed are shown in Tables 1 and 2.

**Table 1:** **Age and gender of children with SARI from whom respiratory swabs were collected or not collected**

**Characteristic**	**Number (Row %)**	
	**Swabbed (N = 815)**	**Not Swabbed (N = 1777)**
**Age**		
<12 m (N = 980)	285 (29.1%)	695 (70.9%)
12-23 m (N = 718)	241 (33.6%)	477 (66.4%)
24-59 m (N = 894)	289 (32.3%)	605 (67.7%)
Total (N = 2592)	815 (31.4%)	1777 (68.6%)
**Gender**		
Male (N = 1369)	411 (30.0%)	958 (70.0%)
Female (N = 1223)	404 (33.0%)	819 (67.0%)

**Table 2:** **Clinical characteristics of children with SARI from whom swabs were collected and not collected**

	**Swabbed (N = 815)**		**Not Swabbed (N = 1,777)**		**P-value**
	**n**	**%**	**n**	**%**	
Cough	806	98.9	1747	98.3	0.91
Difficulty Breathing	435	53.3	575	32.4	0.003*
Unable to Drink or Breastfeed	0	0	2	0.1	0.57
Vomits Everything	155	19.0	338	19.0	0.97
Lethargy	28	3.4	93	5.2	0.53
Chest Indrawing	616	75.6	586	33.0	<0.001*
Stridor	21	2.6	80	4.5	0.47
Oxygen Saturation Measured	808	99.1	1755	98.8	0.99
Oxygen Saturation ≤90%	155	19.0	761	42.8	<0.001*
Temperature ≥ 38°C	447	54.9	492	27.7	<0.001*
Temperature ≥ 39°C	162	19.9	142	8.0	0.02*
Death within 14 days following clinic visit	4	0.5	8	0.5	0.9

*Significant at p < 0.05.

### Household surveillance

Community interviewers visited enrolled households every two weeks and questioned surveillance participants using a standardized questionnaire about recent illnesses, including symptoms and health-seeking, and performed a limited exam on those with symptoms [20]. We defined SARI from the household visits as cough or difficulty breathing with either chest indrawing or elevated respiratory rate for age [20].

### Control selection

From January 1, 2009-February 28, 2011, asymptomatic control-children were enrolled from Tabitha Clinic. Eligible controls were those who presented with non-severe illness (i.e. not requiring hospitalization), for immunizations, or for medicine refills, as described [20]. Eligible controls could not have had fever, any respiratory symptoms or diarrhea during the preceding two weeks. Each month, we attempted to enroll up to six controls <2 years old, and up to six controls 2–4 years old, frequency-matched to cases by known HIV status; however, this target was not met every month. Nasopharyngeal and oropharyngeal swabs were collected from control-children. Ages of controls (mean 2.0, median 1.8) were similar to those of cases (mean 1.7, median 1.5). Controls were not enrolled if there was a SARI case-patient from the same household enrolled on the same day.

### Laboratory testing

Clinic staff trained in phlebotomy collected one to three mL of blood for culture which was inoculated into commercially-produced blood culture bottles. Bacterial growth was identified using standard methodology, described previously (BACTEC™ Aerobic Pedi-PLUS™, Becton Dickinson, Belgium) [28].

Nasopharyngeal and oropharyngeal swabs from cases and controls were combined into 1 ml of viral transport media without antibiotics and transported the same day at 2°C −8°C to KEMRI/CDC laboratories in Nairobi (<30 minutes by automobile), where each specimen was divided into four aliquots and stored at −70°C until testing. Total nucleic acid was extracted from 100 μl of each specimen using MagMAX Viral RNA Isolation Kit (Applied Biosystems) and immediately tested with quantitative real-time reverse transcription polymerase chain reaction (qRT-PCR) assays for adenovirus, respiratory syncytial virus (RSV), human metapneumovirus (hMPV), influenza types A and B viruses, and parainfluenza viruses 1–3 (PIV), as described [20]; A qRT-PCR test result was considered positive if an exponential fluorescence growth curve was detected with an assigned cycle threshold (C_T_)value <40.0 [29,30]. Each clinical specimen was also tested by qRT-PCR for the human ribonuclease P gene to measure nucleic acid integrity and to confirm sample adequacy.

Respiratory swab specimens collected between January 1, 2009 and February 28, 2010 were tested for three additional viruses (rhinovirus, enterovirus, and parechovirus) and atypical bacteria. Total nucleic acid extracts from these specimens were shipped on dry ice from Nairobi to CDC, Atlanta, for testing specifically for the viral pathogens; all other testing throughout the study was done at KEMRI/CDC. qRT-PCR assays for rhinovirus, enterovirus and parechovirus were performed using previously published methodologies [31-33]. The rhinovirus and enterovirus assays target an area of the 5’ noncoding region of an area with high sequence similarity between the viruses that results in some cross-reactivity [34]. Therefore, positive rhinovirus and/or enterovirus qRT-PCR were reported together as rhinovirus/enterovirus positive. For atypical bacteria, multiplex qRT-PCR was performed at KEMRI/CDC laboratories in Nairobi for *Mycoplasma pneumoniae, Chlamydophila pneumoniae* and pan-*Legionella* species (atypical bacteria) using published assays [35]. Neither HIV nor tuberculosis testing was routinely done on children with SARI at the clinic.

### Data analysis

Analyses were performed using SAS (version 9.2, Cary, NC). Proportions were compared using Pearson’s chi-square or Fisher’s exact tests (for small cell counts). Rate ratios and 95% confidence intervals were calculated using Fisher’s method (Computer Programs for Epidemiologists, PEPI, version 4.0x) for crude rates. The Delta method was used for calculating confidence intervals for the adjusted rates taking into account the variation in SARI case numbers, the variation in the adjustment due to clinic visitation, the variation in estimating the proportion of SARI with pathogen detected, and the variation in the pathogen attributable fraction (PAF) estimates (see below) [36].

We compared detection of each virus by qRT-PCR from swabs between cases and asymptomatic controls for the period of January 1, 2009, − Feb 28 2011. Odds ratios (OR) and 95% confidence intervals were calculated using unconditional logistic regression, adjusting for age group (0–11, 12–23, 24–59 months) and season when the swab was taken (December-February, hot and dry; March-May and September-November, the two rainy seasons; and June-August, cool). We used ORs to calculate PAF, which estimates the proportion of cases positive for each virus in which the virus is the likely cause [20,37,38]. The PAFs, calculated as (OR-1)/OR, were only calculated for viruses with ORs that were >1.0. For purposes of this analysis, we assumed the PAF was 1.0 for bacteria detected by blood culture based on the negligible probability of detection of pathogenic bacteria in blood of asymptomatic controls.

SARI incidence was calculated as the number of SARI clinic visits per 100 person-years of observation for the period March 1, 2007-Feb 28, 2011. Revisits for the same illness episode (<7 days respiratory symptom free) were not counted separately. Permanent residence status in the surveillance area was used to determine person-time contribution, as described [39]. Adjusted rates of clinic visitation were calculated accounting for the percentage of all clinic visits made for SARI that were to the Tabitha Clinic (the only clinic in the area where standardized data collection on SARI was done), as opposed to visits to other area clinics, as determined from the household visit interviews [21]. We used any clinic visit as a marker of serious illness, assuming that many illnesses for which no clinic visit was made were likely less severe. Etiology-specific adjusted incidence was calculated by applying the proportions of each etiology to the adjusted incidence of SARI, as shown:

$$ {\mathrm{I}}_{\mathrm{SARI}\mathrm{z}}=\left(\left({\mathrm{Num}}_{\mathrm{SARI}}/\left(\mathrm{S}\mathrm{C}\mathrm{V}/\mathrm{A}\mathrm{C}\mathrm{V}\right)\right)\times \left(\mathrm{Z}/\mathrm{SARI}\right)\times \mathrm{P}\mathrm{A}\mathrm{F}\right)/\mathrm{P}\mathrm{Y}\mathrm{O}\times 100 $$

Where

- I_SARIz_ = Incidence of SARI due to pathogen z (per 100 child-years).
- Num_SARIz_ = number of SARI cases detected at the clinic during the study period.
- SCV/ACV = SARI episodes associated with visiting the study clinic (SARI Clinic Visits or SCV) divided by SARI episodes associated with visiting any clinic (any clinic visits or ACV) (from household visit data).
- Z/SARI = Proportion of SARI cases from which pathogen z is detected.
- PAF = (OR-1)/OR.
- PYO =Person-years of observation.

Etiology-specific incidence was only calculated for pathogens with an OR ≥1.0 in the case–control analysis. We also calculated incidence for pathogens of the same genus or sub-family (i.e. all influenza viruses and all parainfluenza viruses).

We calculated a SARI etiologic fraction (SEF) (also known as population attributable fraction) for each pathogen for which incidence was measured (above) [40,41]. For this analysis, SEF ﻿is the proportion of SARI cases that might theoretically be eliminated if the etiology were eliminated (assuming a primary role for that etiologic pathogen in causing pneumonia). SEF is given as:

$$ \mathrm{S}\mathrm{E}\mathrm{F} = \left(\mathrm{Z}/\mathrm{SARI}\right)\kern0.5em \times \kern0.5em \left(\mathrm{P}\mathrm{A}\mathrm{F}\right) $$

where Z/SARI and PAF are calculated as in the previous equation. To attempt to calculate PAFs adjusting for multiple pathogens (co-infections), we fit a logistic regression model including the pathogens, all pairwise interaction terms for the pathogens, and age and seasonality; the outcome variable for the model was case/control status (1 for case and 0 for control). The RSV and any parainfluenza interaction variable and the RSV and adenovirus interaction variable were borderline significant; all other pathogen interaction variables were non-significant. However, neither of these interaction variables was synergistic in SARI cases (one pathogen being more likely to be found when the other is present). The opposite was true-- the second pathogen was less likely to be found in SARI cases when the other pathogen was present. Therefore, we simply present PAFs, adjusting only for age and seasonality.

### Ethical review

Written informed consent was obtained for data collection at the clinics and households. The protocol and consent forms were reviewed and approved by the Institutional Review Boards of KEMRI (#932) and CDC (#4566).

## Results

A total of 2,592 children <5 years old with SARI were evaluated at the clinic from March 1, 2007-February 28, 2011; 815 (31.4%) had nasopharyngeal/oropharyngeal swab specimens collected. Of these, 34.8% were infants (<12 months old), 29.5% were 12–23 months old, and 35.7% were 24–59 months old (Table 1). Children with SARI who were swabbed were more likely to be febrile and to have difficulty breathing and chest indrawing than children who were not swabbed (Table 2). Among infants < 1 year of age with SARI, the most commonly detected viruses in nasopharyngeal/oropharyngeal specimens were rhinovirus/enterovirus (42%), RSV (25%), adenovirus (20%), and human hMPV (13.7%) (Table 3). In toddlers 12–23 months old, enterovirus/rhinovirus (49%), adenovirus (31.5%). RSV (20.3%) and influenza A (10.8%) were the top four viruses detected, and in children 24–59 months old, the top four pathogens detected were rhinovirus/enterovirus (53%), adenovirus (36.9%), RSV (15.8%), and influenza A (13.4%) (Table 3). Atypical bacteria were not detected in any specimens. Overall, a potential pathogen was not detected in respiratory secretions from 29% of cases.

**Table 3:** **Etiologies of SARI in children < 5 years old, by age group, in Kibera, Nairobi, Kenya**

	**<1 year n (%)**	**12-23 months n (%)**	**24-59 months n (%)**	**All cases, n (%)**
Total patients seen	12445	11838	21385	45668
SARI cases	980 (7.9%)	718 (6.1%)	894 (4.2%)	2592 (5.7%)
Rate of SARI (per 100 PYO)	30.4	16.7	6.7	12.4
CFR^a^ for SARI cases	13 (1.3%)	2 (0.3%)	3 (0.3%)	18 (0.7%)
Blood cultures (BC) done	270 (27.6%)	243 (33.8%)	349 (39.0%)	862 (33.3%)
BCs without contaminant	259 (26.4%)	236 (32.9%)	341 (38.1%)	836 (32.3%)
*S. pneumoniae* ^b^	4 (1.5%)	3 (1.3%)	1 (0.3%)	8 (1%)
*H. influenzae* ^b^	0	0	0	0
*S. aureus* ^b^	10 (3.9%)	1 (0.4%)	3 (0.9%)	14 (1.7%)
*Klebsiella pneumoniae* ^b^	0	0	0	0
Non-typhi *Salmonella* ^*b*^	7 (2.7%)	3 (1.3%)	3 (0.9%)	13 (1.6%)
Salmonella Typhi	1 (0.4%)	2 (0.8%)	2 (0.6%)	5 (0.6%)
Other pathogenic bacteria^b,c^	0	0	1 (0.3%)	1 (0.1%)
Naso/oropharyngeal swabs collected	285 (29.1%)	241 (33.6%)	289 (32.3%)	815 (31.4%)
Influenza A	27 (9.4%)	26 (10.8%)	39 (13.5%)	92 (11.3%)
Influenza B	4 (1.4%)	6 (2.5%)	11 (3.8%)	21 (2.6%)
Influenza A or B	31 (10.9%)	31 (12.9%)	50 (17.1%)	112 (13.7%)
Respiratory syncytial virus	74 (25.9%)	49 (20.3%)	46 (15.8%)	169 (20.7%)
Adenovirus	57 (20%)	76 (31.5%)	108 (36.9%)	241 (29.5%)
Parainfluenza virus 1	10 (3.5%)	10 (4.1%)	8 (2.7%)	28 (3.4%)
Parainfluenza virus 2	7 (2.5%)	7 (2.9%)	11 (3.8%)	25 (3.1%)
Parainfluenza virus 3	31 (10.9%)	27 (11.2%)	19 (6.5%)	77 (9.4%)
Any Parainfluenza	46 (16.1%)	43 (17.8%)	34 (11.6%)	123 (15.0%)
Human metapneumovirus	39 (13.7%)	23 (9.5%)	35 (11.9%)	97 (11.9%)
Rhinovirus/Enterovirus^d^	32/76 (42%)	36/73 (49%)	29/55 (53%)	97/204 (47.5%)
Parechovirus^d^	3/76 (4%)	0	1/55 (1.8%)	4 (2.0%)
Atypical bacteria^e^	0	0	0	0
Positive > =1 virus	204 (71.6%)	182 (75.5%)	195 (66.8%)	581 (71.0%)
Positive > =2 virus	71 (24.9%)	67 (27.8%)	83 (28.4%)	221 (27.0%)

All data presented as number and percentage in parentheses rounded to the nearest integer.

^a^Case-fatality ratios (CFR) are defined as death in the 30 days following clinic visit for SARI episode.

^b^Denominator = uncontaminated blood cultures from 836 patients. Blood culture results discarded if they contained coagulase-negative *Staphylococcus*, *Bacillus species*, or *Corynebacteria*.

^c^Other pathogenic bacterium was *Escherichia coli*.

^d^Rhino/enterovirus, parechovirus, and atypical bacteria were only tested for from January 1, 2009 – February 28, 2010; 204 swabs were tested among persons <5 years old (76 specimens in <1 year olds, 73 on 12–23 month olds and 55 in 24–59 month olds).

^e^presence of atypical bacteria (*Mycoplasma pneumoniae, Chlamydophila pneumoniae*, and *Legionella* species) was assessed by qPCR in 76 np/op swabs.

March 1, 2007-February 28, 2011.

Among 836 uncontaminated blood cultures processed from children with SARI, a pathogen was isolated from 41 (4.9%). *Staphylococcus aureus* (1.7%) was the most common species isolated, followed by non-Typhi Salmonella (NTS) species (1.6%), *Streptococcus pneumoniae* (1%) and *Salmonella* Typhi (0.6%) (Table 3).

When comparing detection rates from respiratory swabs in SARI cases and healthy controls, RSV and influenza A were significantly associated with being a case (Table 4). Several viruses (parainfluenza 1 and 3, and hMPV, influenza B, and adenovirus) were associated with adjusted odds ratios ≥1.5, but the differences between cases and controls were just outside of statistical significance (Table 3). All of the other viruses tested were also found more often in cases than controls with none of these differences approaching statistical significance. Having at least one virus detected from swabs was strongly associated with cases, as was having ≥2 viruses detected (Table 4).

**Table 4:** **Naso/oropharyngeal swab test results from cases and controls**

**Pathogen**	**Cases, n (%) N = 731**	**Controls, n (%) N = 115**	Adjusted OR** (95% CI)
Influenza A	79 (10.8%)	5 (4.3%)	2.57 (1.01-6.52)
Influenza B	19 (2.6%)	1 (0.9%)	3.06 (0.41-23.17)
Influenza A or B	97 (13.3%)	6 (5.2%)	2.71 (1.15-6.39)
Respiratory syncytial virus	155 (21.2%)	3 (2.6%)	10.15 (3.16-32.58)
Adenovirus	221 (30.2%)	27 (23.5%)	1.51 (0.94-2.42)
Parainfluenza virus 1	26 (3.6%)	1 (0.9%)	4.12 (0.55-30.69)
Parainfluenza virus 2	24 (3.3%)	5 (4.3%)	0.71 (0.26-1.9)
Parainfluenza virus 3	72 (9.8%)	5 (4.3%)	2.31 (0.91-5.85)
Any parainfluenza	115 (15.7%)	10 (8.7%)	1.87 (0.95-3.69)
Human metapneumovirus	91 (12.4%)	7 (6.1%)	2.12 (0.96-4.72)
Atypical bacteria	0 (0%)	0 (0.0%)	-
Rhinovirus/Enterovirus *	97 (47.5%)	24 (50.0%)	0.89 (0.46-1.71)
Parechovirus *	4 (1.9%)	1 (2.1%)	0.73 (0.08-6.85)
Positive > =1 virus	533 (72.9%)	61 (53%)	2.27 (1.51-3.42)
Positive >2 viruses	207 (28.3%)	17 (14.8%)	2.36 (1.36-4.11)

*Rhino/enterovirus, parechovirus and atypical bacteria were only tested among a subset of patients. 204 swabs cases were tested among cases <5 years old; 48 controls were tested.

**adjusted for age group (0–11, 12–23, 24–59 months) and season during which the swab was taken.

January 1, 2009, − Feb 28 2011 Kibera, Nairobi Kenya.

Taking the proportion of SARI cases from which a pathogen was detected and the PAF, we calculated the SARI etiologic fraction (see Methods) attributed to viruses or groups of viruses. RSV accounted for the highest etiologic fraction (16.2%) of SARI cases in children <5 years old, followed by adenoviruses (10.6%), parainfluenza viruses (7.0%), and influenza A and B combined (6.7%), and hMPV (6.3%). Since the odds ratios upon which the SARI etiologic fractions were based were significant only for RSV and influenza viruses, the etiologic fractions for the other three pathogens are point estimates (totalling 23.9%) and must be viewed with some caution; however, given an array of co-factors which contribute to the risk of pneumonia, it is conceivable that these pathogens are important in chains of causation. Overall, 46.8% of SARI cases had an independently attributable viral etiology. When blood culture results were included (without regard to blood culture sensitivity), 51.8% of episodes of SARI had an attributable pathogen.

The overall incidence of SARI was 12.4 cases/100 PYO; infants had the highest incidence (30.4 cases/100 PYO). The highest pathogen-specific adjusted incidence rate was with RSV (2.8 cases/100 PYO) (Table 5). The adjusted incidence for influenza A was 1.0 cases/100 PYO and for influenza A and B combined 1.3 cases/100 PYO (Table 5). The rate for parainfluenza virus-associated SARI was 1.1 case/100 PYO and the rates for adenovirus-associated or hMPV-associated SARI were 1.5 and 0.9 case per 100 PYO, respectively.

**Table 5:** **Incidence of select pathogens associated with SARI case status among children < 5, Kibera, Kenya**

**Pathogen**	**% SARI with pathogen detected (from Table** 3 **)**	**Pathogen specific-Number of SARI cases** ^**a**^	**Proportion of all clinic visits for SARI that went to study clinic**	**Adjusted No. of Pathogen specific-SARI cases** ^**b**^	**PAF** ^**c**^	**Final Adjusted Number of Cases** ^**d**^	**Rate/100PYO** ^**e**^ **(95% CI)**
Influenza A	11.2%	290	0.82	354	0.64	227	1.0 (0.4-1.7)
Influenza A and B combined	13.7%	355	0.82	433	0.64	277	1.3 (0.6-2.0)
Respiratory syncytial virus	20.7%	537	0.82	654	0.90	589	2.8 (2.4-3.2)
*S. pneumoniae*	1%	23	0.82	28	1.00	28	0.1 (0.1-0.2)
*S. aureus*	1.7%	41	0.82	51	1.00	51	0.2 (0.2-0.3)
Non-typhi Salmonella	1.6%	39	0.82	47	1.00	47	0.2 (0.2-0.3)
Salmonella Typhi	0.6%	18	0.82	22	1.00	22	0.1 (0.1-0.2)
Influenza B	2.6%	67	0.82	82	0.57	47	0.3 (0.0-0.5)
Adenovirus	29.5%	765	0.82	932	0.36	336	1.5 (0.1-2.9)
Parainfluenza virus 1	3.4%	88	0.82	107	0.77	83	0.4 (0.1-0.7)
Parainfluenza virus 3	9.4%	244	0.82	297	0.55	163	0.8 (0.2-1.4)
Any parainfluenza	15.0%	389	0.82	474	0.46	218	1.1 (0.2-1.9)
Human metapneumovirus	11.9%	308	0.82	376	0.53	199	0.9 (0.3-1.6)

^a^Pathogen-specific number of cases = % SARI (with swabs collected) with specific pathogen detected multiplied by 2,592—i.e. the number of overall SARI cases (with and without swabs collected).

^b^Adjusted number of pathogen-specific SARI cases = Pathogen specific number of SARI cases/0.82 (Proportion of all clinic visits for SARI that were to study clinic).

^c^PAF = Pathogen Attributable Fraction (see methods).

^d^Final adjusted number of cases = Adjusted No. of pathogen specific-SARI cases multiplied by PAF.

^e^Rates calculated per 100 person-years of observation.

March 1, 2007-February 28, 2011.

**Figure 1:**
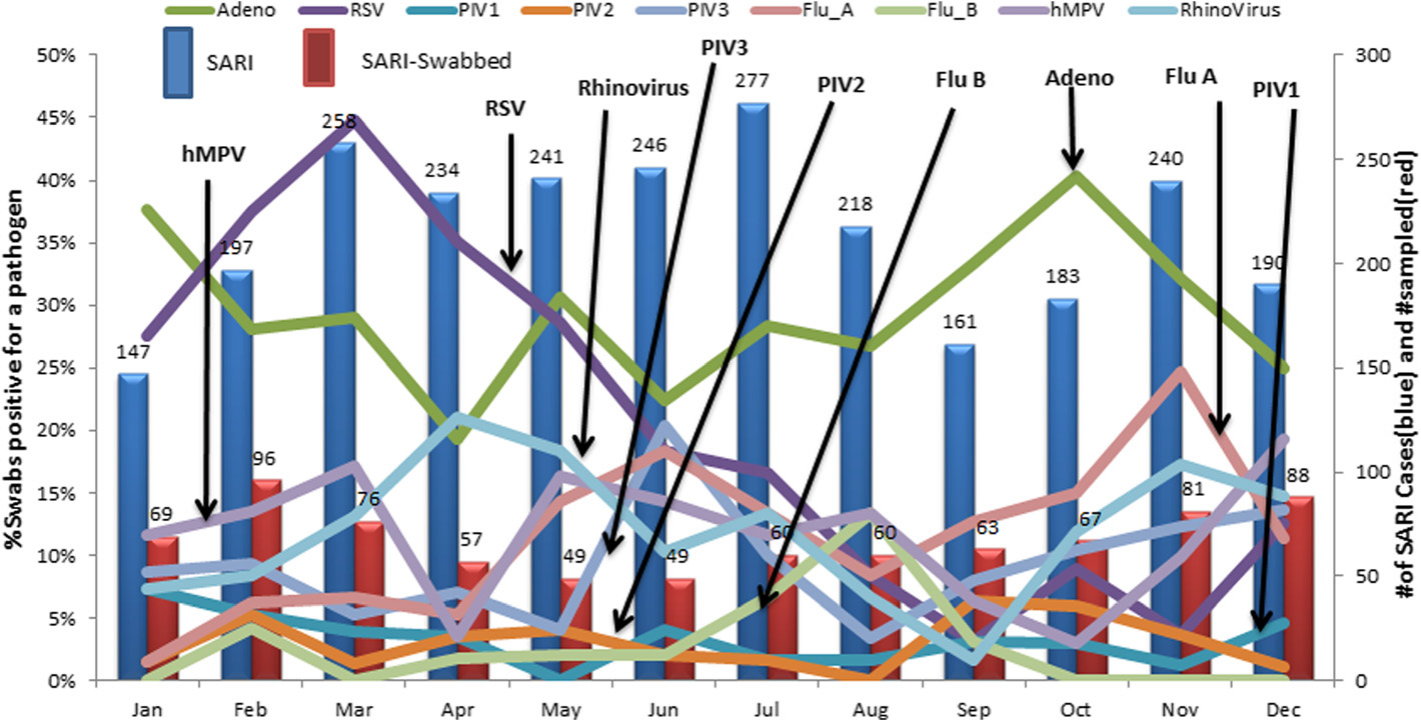
**Monthly (aggregate) distribution of viral pathogens, March 1, 2007-Feb 28, 2011.**

During the four year study period, RSV primarily occurred during January through April (peaking in March and April) (Figure 1), a warm and dry, pre-rainy season in Nairobi. Influenza A was detected year round with a peak from October through December, a rainy season, and a smaller peak in June and July, the cold season. While adenoviruses were detected year round, two peaks occurred, from January through March, and September through December. Other pathogens were detected at low frequency every month.

## Discussion

This study characterizes the incidence of major respiratory viral pathogens as causes of SARI in children. Because of insensitivity of diagnostic tests for bacteria, the roles of key bacterial etiologies were likely underestimated. The rates for SARI are lower than what we observed in a similar study conducted over the same time interval in a rural, sparsely populated area of Kenya (Lwak) about 250 km away by road, which also found that influenza A and RSV were significant contributors to SARI (20). Both areas have high HIV seroprevalence in adults (15-20%) (24), but the rural area is holoendemic with malaria (which can present as SARI [42]), whereas in Kibera local transmission of malaria is felt to be low.

The case definition we used for SARI is similar to definitions used for severe and very severe pneumonia by WHO and for ALRI, the only difference being that we added a clinical criterion of oxygen saturation <90% to add some specificity for severe disease. Thus, the incidence data from this study should be interpretable within the context of other reports of severe and very severe pneumonia and of acute lower respiratory infection in children with the caveat that the study clinic only serves outpatients, referring severely ill children to a nearby hospital; therefore, the clinical spectrum likely differed from studies of hospitalized SARI patients. However, children with signs of severe illness (like chest indrawing, difficulty breathing and high fever) were more likely to be sampled while those with low oxygen saturation were not.

Decades of research have consistently found RSV to be the most important viral pathogen causing significant acute respiratory illness in infants and young children [2,5,10,43]. A recent meta-analysis of published and unpublished data found that the incidence of RSV-associated severe ALRI has ranged from 10–18 per 1000 children/year for infants and 3–9 per 1000 per children <5 years of age/year in Africa, with most data coming from passive, hospital-based surveillance with defined catchment areas [44]. These rates were much lower than those found in Alaska and among the Navajo and White Mountain Apache population in the US and in a site in Guatemala [44]; it is unclear whether the differences are due to ecological variations or methodological differences. The rates from Kibera for RSV-associated SARI, resulting from active, community-based surveillance, which made it possible to account for cases missed during clinic surveillance, were slightly higher than the rates from the earlier African studies, but lower than the rates we found in the parallel rural study [20]. Our rural study clinic included in-patients, which increased the likelihood of including sicker patients, than in Kibera, where very sick patients may have chosen to go directly to hospital. The rates from both studies likely underestimate RSV (and other SARI) incidence because we only adjusted for that portion of patients who went to a clinic other than the study clinic; we did not adjust for under-utilization of any clinical services, which is a factor to consider in both the urban and rural sites [17,18].

Over the past decade, the number of studies of influenza has accelerated in sub-Saharan Africa [45]; data from 15 countries from 2006–2010, including Kenya (but not Kibera) showed that (without controls) 10% of SARI cases <5 years old had evidence of influenza infection [46], which was similar to what we observed in Kibera. A meta-analysis of published and unpublished global data, including 2008 data from the Kibera site (among many others), found a rate of influenza associated ALRI of 14/1000 children <5 years old in developing countries [47], imputing that 111,500 deaths due to influenza (range = 21,000-245,000) occurred in 2008. As documented in other tropical environments [48], influenza in Kibera appeared to be a year-round phenomenon without a predominant occurrence in cool months.

Specimens from cases alone would have suggested that rhinoviruses were the most important viruses for SARI in all three age groups; however, rhinoviruses were detected more often from control specimens. This study does not rule out a role for human rhinoviruses in SARI, however, as nasopharyngeal specimens are not consistently the optimal sample for determining the cause of lung pathology. A recent outbreak of fatal respiratory infection in infants in Vietnam was attributed to a subclade of a genotypically distinct cluster of rhinoviruses [49], and earlier reports have documented association of specific genotypes of rhinoviruses with ALRI [50,51]. We did not assess the genotype of the rhinoviruses detected in this study to determine if there were specific rhinovirus clusters associated with illness when compared with controls.

*M. pneumoniae* and *C. pneumoniae* were not detected in any specimens; their role in SARI in children in the developing world is unclear. A recent study from Madagascar detected *M. pneumoniae* and *C. pneumoniae* within a small subset of children with respiratory illness, without a healthy control group for comparison [52] and a prospective study in Tunisia suggested that *M. pneumoniae* was the cause of >7% of hospitalized pneumonias in children [53].

While our studies and others have consistently shown that influenza A and RSV are important contributors to SARI in children, our data also suggest that when considered together, PIV, adenovirus and hMPV play a causal role for approximately 24% of SARI cases with the caveat that statistical significance when comparing cases and controls was not quite achieved for these viruses. The 3 viruses are likely important pathogens to consider when developing technologies and packaging strategies to prevent severe acute respiratory illness. However, it should be noted that because of frequent adenovirus infection in early childhood and persistent shedding, many detections of adenovirus by rRT-PCR could represent prevalent rather than incident infections [54].

There appeared to be a broader range of viruses causing SARI in Kibera when compared with our rural site in Lwak [20] and with a recent study conducted in rural Kilifi, Kenya, which also only found significantly greater detection of RSV when comparing pneumonia cases with controls [55]. In our rural study, which was conducted for a shorter, overlapping interval, all odds ratios were ≤1.3 (with wide confidence intervals) for each of the same viruses tested as in Kibera, except for the odds ratio associated with infection with PIV-2 (OR = 2.6;95% CI =0.6-10.0). While it is tempting to hypothesize that the population density in Kibera when compared to the rural site (300 people/km^2^) provides a more dynamic “melting pot” for virus introduction and spread, we have insufficient additional evidence to support the notion.

We used blood culture to document invasive bacterial infection in patients with SARI. The highly specific results are, nonetheless, very insensitive. No sensitive tests for *S. pneumoniae* and other bacterial pathogens are currently available. However, historical data and vaccine probe studies [56] have shown that a substantial proportion (10-20%) of SARI was likely due to pneumococcal infection; thus, the rate we found (shown in Table 3) substantially underestimates the rate of *S. pneumoniae*-associated SARI. Since Hib vaccine is given routinely in Kenya, Hib was likely not an important contributor to SARI incidence. We detected NTS and *Salmonella* Typhi in blood culture from only a few patients. However, given the importance of NTS and *S.* Typhi as causes of acute febrile illness often with respiratory symptoms, in some settings [39,57], it is worthwhile to be aware of their potential role. The impact of these pathogens may change, especially with urbanization and growth in slums, which especially appears to increase the risk for typhoid fever [39]. The data from this study are consistent with a study from South Africa suggesting that *S. aureus* may be an important cause of pneumonia in children [58]; in that study, there was a potential link to HIV infection. In an urban setting in Nigeria, *S. aureus* was the most common cause of bacteremia in children with pneumonia [59]. More data are needed to characterize the burden, co-morbidities and other risk factors for children with *S. aureus* bacteremia-associated SARI, recognizing the potential that some of the microbiological results may represent contamination.

By calculating etiologic fractions for SARI, approximately one-half of SARI cases are accountable with the pathogens we studied. If one assumes, based on pneumococcal vaccine probe data [56,60] that approximately 25% of SARI (based on a 12% reduction in severe pneumonia and a 50% vaccine efficacy against all pneumococcal serotypes from vaccine studies) is due to *S. pneumoniae*, then up to 75% of SARI episodes might be attributable to a specific etiologic agent. Thus, we could not attribute illness to a specific pathogen in at least 25% of cases. The most likely explanation for this diagnostic gap is insensitivity of diagnostic tests and type of specimens-- nasopharyngeal and oropharyngeal swabs are not the most efficient techniques for capturing all respiratory viruses [61] and are not appropriate for detecting key bacterial etiologies of pneumonia. Timing of specimen collection vis-à-vis peaks in virologic shedding may also have played role. It is also likely that pathogens other than the ones we tested for (possibly including pathogens not yet known) caused a proportion of these illnesses, and will become important public health targets in the future as new diagnostic tests become available and, also, potentially as new pathogens are recognized. In addition, clinical syndromes, like malaria, can masquerade as pneumonia, thus accounting for a proportion of the “non-attributable causes.”

There were a number of limitations associated with the findings of this investigation. We were limited in this analysis by the small number of controls. This restricted the precision of our estimates of pathogen attributable fraction; consequently, we could not confirm the precise role of several pathogens that may be important contributors to the burden of SARI. In addition, quantifying the viral load in respiratory secretions (which we did not do in this analysis) could potentially identify a threshold of pathogen load associated with illness versus asymptomatic colonization [62,63]. Also, we did not test for other pathogens that might contribute to the incidence of SARI, like human coronaviruses, bocaviruses, and others [64-66]. The subset of children with SARI who were swabbed were more likely to have several indicators of more severe illness (like chest indrawing) than children with SARI who were not swabbed, although low oxygen saturation was more often observed in children who were not swabbed. Thus, incidence rates of pathogens which are linked to specific clinical presentations may have been overestimated. For calculating pathogen-specific incidence rates of SARI, we used one adjustment factor, based on the proportion of all clinic visits for SARI that were to the study clinic, because there was no available approach to make this adjustment by etiology; however, in reality, study clinic visitation patterns may not have been consistent across etiologies, since some may be associated with symptoms more likely to induce a visit to the study clinic, rather than other clinics in the area. Finally, since our case definitions for SARI did not include apnea, which can be a severe manifestation of RSV-associated bronchiolitis in neonates, we likely underestimated the role of RSV in young infants. Furthermore, the active case detection, and enhancements within the community to encourage early treatment, may have reduced mortality from SARI, and also reduced detection of more severe forms of the syndrome, probably led to underestimations of incidence, especially for pathogens most likely to result in greater clinical severity of illness; thus, our findings may not be representative of SARI cases studied elsewhere.

## Conclusions

The findings from this study suggest that interventions focused on preventing influenza A, RSV, and, perhaps a combination of parainfluenza viruses, adenovirus and human metapneumoviruses may have substantial impact on further reducing the burden of respiratory infections in children in developing countries, adding to the expected impact of pneumococcal conjugate vaccines, which are in the process of being introduced in lower income countries in Africa and Asia, and expected to diminish pneumococcal disease burden [67]. For influenza, a variety of tools (vaccines and strategies to deliver them) already exist [68,69]. For several other key viral pathogens, vaccine development continues to progress [70,71]. Non-etiology-specific interventions like improving hand hygiene, reducing exposure to smoke in the home [72,73], and making oxygen available in clinical settings will likely contribute to reducing severe illness and mortality.
